# Micro- and Macro-Algae Combination as a Novel Alternative Ruminant Feed with Methane-Mitigation Potential

**DOI:** 10.3390/ani13050796

**Published:** 2023-02-22

**Authors:** Eslam Ahmed, Kengo Suzuki, Takehiro Nishida

**Affiliations:** 1 Department of Life and Food Sciences, Obihiro University of Agriculture and Veterinary Medicine, Inada, Obihiro 080-8555, Japan; 2 Department of Animal Behavior and Management, Faculty of Veterinary Medicine, South Valley University, Qena 83523, Egypt; 3 The Research and Development Department, Euglena Co., Ltd., Tokyo 108-0014, Japan

**Keywords:** *Asparagopsis taxiformis*, digestibility, euglena, feed additives, global warming, nutritive value, rumen fermentation

## Abstract

**Simple Summary:**

Considering the current challenges facing the modern livestock industry and the food insecurity situation, there is an urgent need to find alternative, sustainable, climate-friendly, and safe feed ingredients. This study provides a novel solution with the mixture of *Euglena gracilis* and *Asparagopsis taxiformis* as a feed for ruminants. *Euglena gracilis* is a highly nutritive material that can be used to partially replace the expensive, high-quality ingredients in the diet. Due to its bromoform content, *Asparagopsis taxiformis* is efficacious in reducing methane emissions. However, there are some health concerns for animals and humans with regard to its usage, as well as some doubts about the mass production that is required to achieve effective methane reduction. Therefore, the current study evaluated a new formulation composed of the minimum effective levels of *Euglena* and *Asparagopsis* to partially replace the concentrate mixture in the ruminant diet and reduce methane emissions. This combination had a synergistic effect in reducing methane production that was better than supplementing these algae individually and had no adverse impacts on animal productivity indices. Therefore, this intervention has double-sided benefits, providing high-quality alternative feed and reducing methane emissions with lower amounts of *Asparagopsis*.

**Abstract:**

This study was conducted to provide alternative high-quality feed and to reduce methane production using a mixture of the minimum effective levels of *Euglena gracilis*, EG, and *Asparagopsis taxiformis*, AT. This study was performed as a 24 h in vitro batch culture. Chemical analysis demonstrated that EG is a highly nutritive material with 26.1% protein and 17.7% fat. The results showed that the supplementation of AT as a feed additive at 1 and 2.5% of the diet reduced methane production by 21 and 80%, respectively, while the inclusion of EG in the diet at 10 and 25% through partially replacing the concentrate mixture reduced methane production by 4 and 11%, respectively, with no adverse effects on fermentation parameters. The mixtures of AT 1% with both EG 10% and EG 25% had a greater reductive potential than the individual supplementation of these algae in decreasing methane yield by 29.9% and 40.0%, respectively, without adverse impacts on ruminal fermentation characteristics. These results revealed that the new feed formulation had a synergistic effect in reducing methane emissions. Thus, this approach could provide a new strategy for a sustainable animal production industry.

## 1. Introduction

Modern livestock systems are challenged by greenhouse gas (GHG) emissions, land degradation, and feed costs [1,2]. The livestock industry, particularly the ruminant production sector, plays an important role in food security because it is capable of converting indigestible plant mass to meat and milk, which are sources of high-quality protein and essential nutrients for human well-being [3,4]. However, this industry accounts for approximately 14–18% of global anthropogenic GHG emissions, of which enteric methane (CH_4_) production is the most significant source because its global warming potential is 28 times greater than that of carbon dioxide (CO_2_) on a 100-year timescale [5]. Moreover, livestock occupies two-thirds of the world’s agricultural land, either for grazing or crop cultivation [6]. In addition, the production of conventional feed protein concentrates such as soybean is a very expensive process and may be viewed as being in direct competition with human food security because they are considered staple food crops for humans [7]. On the basis of the aforementioned challenges, in addition to the rise in the global population and increased demand for animal-derived products, finding natural alternatives and high-quality feeds is of great importance for a sustainable animal production industry. These novel feeds may replace the expensive conventional sources in animal diets and help reduce the environmental impact of the animal husbandry sector. Algae are one of the suitable options that have that potential. Algae are classified into two categories: microalgae, which are microscopic, small unicellular organisms that are commonly referred to as phytoplankton, and macroalgae, which are multicellular and can be of very large size; these are commonly known as seaweeds. 

Microalgae supplementation is one of the prospective solutions as a promising alternative source of nutrition for cattle. Microalgae are photosynthetic microorganisms that consume CO_2_ via light energy to produce a variety of proteins, carbohydrates, and lipids [8,9]. The nutritional profiles of microalgae species are quite variable; however, the majority are characterized by protein (25–40%), fat (10–30%), and carbohydrate (5–30%) contents that are comparable, if not superior, to traditional feed ingredients like soybean meal, corn, and wheat [10,11,12]. Recent studies have shown that the inclusion of microalgae can improve the growth performance of animals for both meat and milk production [13,14]. One example is *Euglena gracilis* (EG), a freshwater microalga that is characterized by its high protein and fat content [15]. A recent study conducted with Holstein calves has shown that the supplementation of EG β-glucans to the milk replacer improved their growth performance and ameliorated diarrheal bouts [16]. Studies on the effect of EG as a feed, considering its impact on ruminal fermentation and CH_4_ production, are scarce. In an in vitro study, the inclusion of up to 10% of EG to replace concentrates in the ruminant diet reduced CH_4_ production by up to 9% owing to the effect of fatty acids, without negative impacts on the fermentation profile [17]. The EG is a commercially available product from Euglena Co., Ltd., Japan. This company has its own facilities at Ishigaki Island, Japan, for the large-scale cultivation and production of EG [18]. Further research and innovation could improve the utilization of EG as a sustainable alternative feed with greater CH_4_-reduction potential.

Recently, macroalgal seaweeds have emerged as a potential dietary strategy to reduce CH_4_ emissions from ruminants [19]. The most effective candidate is the red seaweed, *Asparagopsis taxiformis* (AT). With an inclusion rate of 1–3%, AT can reduce CH_4_ production by more than 80% because of its main active component, bromoform [20,21]. Bromoform has been shown to react with vitamin B12, which hinders the cobamide-dependent methyltransferase reaction, a terminal step in CH_4_ formation [22]. Despite its strong reduction potential, AT also has high concentrations of heavy metals and minerals, such as iodine, which can be excreted in milk, leading to undesirably high levels in animal products, which can have adverse health impacts on humans [23,24]. Notably, there are human safety concerns regarding the main active component of AT (bromoform) present in milk [25]. Additionally, one study showed that the supplementation of sheep with AT led to pathological changes in the ruminal mucosa [21]. However, the inclusion of low doses of AT in feed formulations can reduce these risks in both animals and humans, but this also diminishes the mitigation potential to reduce CH_4_ production [26,27]. Another challenge is that the amount of AT required to be supplemented in ruminant feed (50–100 g/day for cattle) is tremendous. There is currently a growing interest in investing in that area through many startup companies such as CH4 Global™, Inc. in Australia (CH4 Australia PTY Limited) and New Zealand (CH4 Aotearoa Limited)to ensure constant large-scale production. The company announced the first commercial supply of its proprietary AT-based product for ruminant animals in June 2022. However, the mass production needed to feed ruminants on a larger scale is currently infeasible [27,28].

Therefore, considering the advantages of EG and AT, the main purpose of the present study was to formulate a new mixture composed of the minimum effective levels of EG and AT to reduce CH_4_ emissions without adverse impacts on ruminal fermentation. The selected dosages for AT and EG in the current study were based on previously conducted pre-trials. In this experiment, the minimum effective levels of EG (as a feed) and AT (as a feed additive) were used in different combinations to study the impacts on CH_4_ production and ruminal fermentation characteristics. We hypothesized that the new feed formulations (EG as a feed + AT as a feed additive) would efficiently substitute part of the concentrate mixture in the diet with EG and reduce CH_4_ production in an additive or synergistic manner without negatively affecting ruminal fermentation.

## 2. Materials and Methods

This study was approved by the Animal Care and Ethics Committee of the Obihiro University of Agriculture and Veterinary Medicine, Japan (approval number 21-41). The animals involved in these experiments were kept and cared for by the Field Science Center. Animal management and sampling procedures were performed according to the established guidelines at the Obihiro University of Agriculture and Veterinary Medicine. The collection of seaweeds and experimental research complied with institutional, national, and international guidelines and legislation. The in vitro experiments were conducted during the period from 8 July to 18 September 2021. This study followed the standard procedures and is reported in accordance with ARRIVE guidelines.

### 2.1. Experimental Feeds

Kleingrass (*Panicum coloratum*) hay and a commercial concentrate mixture (the composition of the ingredients is presented in Table 1) were used as the basal diet. They were ground using the Retsch SM-2000 cutting mill (Retsch, Germany) to pass through a 1 mm sieve. The proximate analyses of the grass hay and concentrate mixture are presented in Table 1. The tested material of EG in fine powder form (10–150 μm particle size) was obtained from Euglena Co., Ltd., Japan. More details about EG growth, mass cultivation, and processing were described previously [18,29]. The tested material of EG in this study was characterized by high protein (26.1%), fat (17.7%), and carbohydrates (51.2%) (Table 1). Approximately 35% of the carbohydrate content in our material was paramylon. The tested material had all the essential amino acids (Table 2). The EG was rich in saturated fatty acids (59.7%), whereas the unsaturated fatty acid content was 34.3% (Table 3). The biomass of AT was harvested during the gametophyte phase at Kochi Bay, Kochi City, Kochi Prefecture, Japan on 25 May 2021 by Dr. Takuma Mezaki. The harvested biomass was stored on ice and shipped to the Obihiro University of Agriculture and Veterinary Medicine. Once the AT was received, it was stored at −20 °C until further processing. The biomass was kept at 4 °C overnight to thaw, then it was dried in an oven dryer at 45 °C for 24 h and then ground by a cutting mill (Retsch SM-2000, Haan, Germany) to pass through a 1-mm sieve on 05 July 2021. The ground material was packaged in aluminum laminated film zipper bags (Lamizip^®^, AL-16, Seisan Nipponsha, Ltd., Tokyo, Japan) and kept at −20 °C until use. The OM content of the AT was 41.7% (Table 1), while the bromoform concentration in the AT was 953.8 μg/g DM.

### 2.2. Donor Animals and Rumen Fluid Collection

Approximately 1.3 L of rumen fluid was collected 3 h after morning feeding from two ruminally cannulated non-lactating Holstein cows that were approximately 8 years old with an average body weight of 894 kg. The cows were fed at the maintenance level on a diet of orchard grass (*Dactylis glomerata*) hay (organic matter (OM), 980 g/kg; crude protein (CP), 132 g/kg; neutral detergent fiber (NDF), 701 g/kg; acid detergent fiber (ADF), 354 g/kg; acid detergent lignin (ADL), 40 g/kg; dry matter (DM) basis) with free access to clean drinking water and mineral blocks (Koen^®^ E250 TZ, Nippon Zenyaku Kogyo Co., Fukushima, Japan). The collected rumen fluid was strained through four layers of surgical gauze, placed into a thermos flask that had been pre-warmed to 39 °C, and then immediately transferred to the laboratory within 15 min.

### 2.3. Experimental Design

This study was conducted as an in vitro batch culture according to the procedure described by Menke and Steingass [30]. This experimental design was composed of nine treatments: control (50% hay:50% concentrate), two inclusion levels of AT as a feed additive (1 and 2.5% of the basal diet), two inclusion levels of EG as a feed in the basal diet (10 and 25%) partially replacing the concentrate mixture, and four different formulations as a combination of EG (as a feed) and AT (as a feed additive) as follows: EG 10% + AT 1%, EG 10% + AT 2.5%, EG 25%+ AT 1%, and EG 25% + AT 2.5%. Each treatment had four replicates, and this experimental design was repeated in three separate runs in different weeks. Two bottles were used as blanks in each run.

### 2.4. In Vitro Incubation and Sample Collection

Approximately 500 mg of substrate (basal diet) was added to pre-weighed nylon bags with a pore size of 53 ± 10 μm (BG1020, Sanshin Industrial Co., Ltd., Kanagawa, Japan), which were heat-sealed and placed in 120 mL glass bottles. Since the AT was applied as a feed additive, it was added directly to the bottles, while the EG was evaluated as a feed, so it was added to the nylon bags. Under continuous CO_2_ flushing, 40 mL of fresh buffer solution (pH 6.8) prepared according to McDougall [31] and 20 mL of the collected rumen fluid were added to each fermentation bottle. The bottles were then flushed with CO_2_ before being sealed with butyl rubber stoppers and aluminum caps (Maruemu Co., Ltd., Osaka, Japan). All the bottles were incubated for 24 h at 39 °C.

At the end of the incubation, the total gas production was measured using a calibrated syringe, and an aliquot of the headspace gas was collected from each bottle and stored in a vacutainer tube (BD Vacutainer, Becton Drive, NJ, USA) at room temperature until CH_4_ and CO_2_ were determined by means of gas chromatography (GC-8A, Shimadzu Corp., Kyoto, Japan), as described previously by Ahmed et al. [32]. Then, the pH was determined, and 1 mL of the culture medium was collected in Eppendorf tubes (Eppendorf AG, Hamburg, Germany) and centrifuged at 16,000× *g* at 4 °C for 5 min. The supernatant was used to estimate the volatile fatty acids (VFA) by means of high-performance liquid chromatography (HPLC, Shimadzu Corp., Kyoto, Japan) and ammonia nitrogen (NH_3_-N) by using a microplate reader (SH-1000 Lab, Corona Electric Co., Ltd., Hitachinaka, Japan), as processed and reported previously [32]. The nylon bags were rinsed with tap water until the effluent became clear, after which they were dried at 60 °C for 48 h and weighed to determine the in vitro dry matter digestibility (IVDMD) [33]. The IVDMD was calculated as the DM that disappeared from the initial DM weight input into the bag.

### 2.5. Chemical Analysis

The chemical compositions of grass hay, concentrate, EG, and AT were determined according to AOAC standard procedures [34]. The DM content was determined by drying the samples in an air-forced oven at 135 °C for 2 h (method 930.15). The OM was measured by placing the samples in a muffle furnace at 500 °C for 3 h (method 942.05). The ether extract (EE) was determined according to method 920.39 while nitrogen was measured according to the method of Kjeldahl (method 984.13) using an electrical heating digester (DK 20, VELP Scientifica, Usmate (MB), Italy) and an automatic distillation apparatus (UDK 129 VELP Scientifica, Usmate (MB), Italy). The CP was then estimated as nitrogen × 6.25. The NDF, ADF, and ADL were estimated and expressed as inclusive residual ash using an ANKOM^200^ fiber analyzer (Ankom Technology Methods 6, 5, and 8, respectively; ANKOM Technology Corp., Macedon, NY, USA) [35]. The NDF was measured using sodium sulfite without the heat-stable α-amylase. The non-fiber carbohydrate was estimated according to the following formula: 100 − (NDF %+ CP %+ EE % + Ash %). The chemical compositions of the experimental treatments are presented in Table 4.

### 2.6. Bromoform Concentration in Asparagopsis

The analysis of bromoform was conducted using a gas chromatograph mass spectrometer (GCMS-QP2010Plus, Shimadzu Corp., Kyoto, Japan) combined with a TurboMatrix HS40 headspace sampler (PerkinElmer, Waltham, MA, USA) and equipped with an RTX-624 capillary column (60 m × 0.32 mm i.d.; film thickness 1.8 μm; Restek, Bellefonte, PA, USA). The column temperature was programmed to be maintained at 35 °C for 5 min, increased to 230 °C at a rate of 10 °C/min, and held at 230 °C for 5 min. The detector temperature was set at 200 °C. Pure helium was used as the carrier gas. The internal standard was a fluorobenzene stock solution (Kanto Chemical Co., Inc., Chuo-ku, Tokyo, Japan). This analysis was performed at a private institute (Zukosha Co., Ltd., Obihiro, Japan).

### 2.7. Fatty Acid and Amino Acid Composition of Euglena

The fatty acid and amino acid profiles of EG were analyzed by the Japan Food Research Laboratories, Japan. The fatty acid composition was determined using gas chromatography (7890 B, Agilent Technologies, Inc. Wilmington, DE, USA) equipped with a flame ionization detector. Each fatty acid was calculated on the basis of the standard retention time and was reported as a percentage of the total fatty acid content. The amino acid composition, except for tryptophan, histidine, phenylalanine, and leucine, was determined using an automated amino acid analyzer (JLC-500/V, JEOL Ltd., Tokyo, Japan; column, LCR-6 with 4 mm × 120 mm ID, JEOL, Co., Ltd., Tokyo, Japan). Tryptophan was analyzed using HPLC (LC-40D, Shimadzu, Co., Ltd., Kyoto, Japan). The column was CAPCELL PAK C18 AQ (4.6 mm ID × 250 mm, Osaka Soda Co., Ltd., Osaka, Japan) with fluorescence detection (RF-20Axs, Shimadzu, Co., Ltd., Kyoto, Japan). Histidine, phenylalanine, and leucine were analyzed using HPLC (LA8080, Hitachi High-Tech Corporation, Tokyo, Japan) on a column packed with Hitachi custom ion exchange resin (4.6 mm ID × 60 mm, Hitachi High-Tech Corporation, Tokyo, Japan). Each amino acid was reported as a percentage of the total amino acid composition. Further information on the analytical specifications for both fatty acids and amino acids was previously provided by Ahmed et al. [36].

### 2.8. Statistical Analysis

The data were screened for normality using the PROC UNIVARIATE of SAS statistical software version 9.4 (SAS Institute Inc., Cary, NC, USA). Then, data were analyzed using PROC MIXED with models including the treatments as a fixed effect, whereas the three experimental runs were considered random effects. Orthogonal polynomial contrasts were conducted to test the linear and quadratic responses to increasing levels of EG and AT. Comparisons of AT versus AT + EG (all dosages), EG versus AT + EG (all dosages), and AT 1% versus AT 1% + its combination with EG at 10 and 25% were conducted as well. The values are shown as means with pooled standard errors of the means. Differences in the means among the experimental groups were estimated using Tukey’s test. Differences were considered statistically significant at *p* < 0.05.

## 3. Results

The yield (mL/g) of CH_4_/DM significantly decreased by 21.3 and 80.1% after supplementation with 1 and 2.5% AT, respectively, compared to the control group (*p* < 0.01, Table 5). The inclusion of 10 and 25% EG in the diet decreased the CH_4_ yield by 4.4 and 11%, respectively; these findings were not significant (*p* > 0.05, Table 5) when compared with the control group. The combination of 1% AT and 10% EG in the diet led to a significant CH_4_ reduction potential of up to 29.9% compared to the control (*p* < 0.01, Table 5). A more reductive potential of up to 40.1% was observed with the combination of 1% AT and 25% EG (*p* < 0.01). In contrast, the combination of 2.5% AT with neither EG (10%) nor EG (25%) was effective in achieving a more significant reduction than individual supplementation of 2.5% AT (75.9 and 81.7% versus 80.2%, respectively, *p* = 0.09, Table 5).

The fermentation characteristics in Table 6 show that the combination of 1% AT with 10% EG or 25% EG had no adverse effect on ruminal fermentation parameters or IVDMD. These combinations shifted the fermentation profile towards more propionate and less acetate (*p* < 0.01). The combination of 2.5% AT and 25% EG led to a decrease in the production of total VFA (*p* < 0.01). The concentration of NH_3_-N was higher (*p* < 0.05) with the inclusion of 25% EG, either individually or in combination with 1 and 2.5% AT, compared to the basal diet in the control group.

## 4. Discussion

To the best of our knowledge, this is the first study to evaluate the combination of EG and AT as a novel sustainable feed for ruminants. It is important to mention that in vitro studies are a valuable tool for the study of ruminal CH_4_ production; however, any approach needs to be investigated with animals as well.

The chemical composition analysis of EG performed in this study confirmed the previous findings of Aemiro et al. [17], who reported that EG is a rich source of amino and fatty acids. Aemiro et al. [12] reported that EG has an essential amino acid profile very similar to that of soybean. The EG was also characterized by very low NDF content because it does not have a carbohydrate-based cell wall such as that found in terrestrial plants [15,37]. It is noteworthy to mention that the carbohydrate storage in microalgae is different from that in vascular plants (e.g., grasses or herbs), and additionally, there is huge variation between different microalgae genera; it may even vary from species to species within the genera [15]. The carbohydrate storage in EG is known as paramylon (classified as dietary fiber), an insoluble, highly crystalline, fibrillary β-(1,3) linked glucan [38]. Paramylon crystallinity is comparable to that of cellulose [39]. Therefore, the rumen microbiome might need some time to adapt to this carbohydrate mixture as a nutrient source.

The ash content of the AT in the current study is relatively high (greater than 50%), which is the same as that which was observed previously [21,25,40,41]. According to the previous studies where the nutritional value was evaluated, it was reported that the main elements that formed the major basis of ash were sodium and potassium [41,42,43,44]. The bromoform concentration of the tested AT in the current study (953 μg/g DM) was much lower than that reported in other studies where the AT was collected from the Santa Catalina island, 35 km off the coast of southern California, United States (dried at 55 °C for 72 h, bromoform 2305 μg/g DM [43]), and Humpy island, Keppel Bay, Queensland, Australia (freeze-dried, bromoform 6550 μg/g DM [20], and 7800 μg/g DM [45]). The lower concentrations in our study can be attributed to the processing methods used. Vucko et al. [40] found that drying the AT biomass at 45 °C led to a substantial reduction in bromoform concentration. This could explain the lower concentration of bromoform in our study, because the AT was dried at 45 °C for 24 h. Additionally, the concentration of bioactive components in seaweeds can vary across seasons and geographical locations [24,46], which might explain why the bromoform in the present study was lower than that in California, United States, where the authors dried AT 55 °C for 72 h [43].

The current study showed that AT had a dose dependent potency in reducing CH_4_ production, as previously reported [47]. This effectiveness has been linked to the halogenated bioactive compounds present in AT, such as bromoform, dibromochloromethane, bromochloroacetic acid, dibromoacetic acid, and dichloromethane [48]. Bromoform is the most abundant compound in AT and has been shown to inhibit enzymatic activity by binding to vitamin B_12_ that chemically resembles coenzyme F_430_, a cofactor required by methyl-coenzyme M reductase, which catalyzes the last step of CH_4_ synthesis [22,49].

It is well established that the addition of dietary fat, regardless of its source, can reduce CH_4_ production by approximately 2.2–5.6% [50,51]. The potency of EG in reducing CH_4_ in this study could be attributed to its high fat content. One possible theory is that fat reduces the fermentation of organic matter, thus decreasing the available hydrogen for methanogens [52]. However, the unsaturated fatty acid content in EG may have a direct role in reducing CH_4_ production through bio-hydrogenation as an alternative hydrogen sink [53]. In addition, the saturated fatty acids, such as myristic and palmitic, which are abundant in EG, could have played a significant role in the reduction potential by increasing the cell membrane permeability of microorganisms associated with methanogenesis [54].

Accordingly, the combination of different modes of action represented by EG and AT led to a synergistic effect in inhibiting methanogenesis, which was obvious with the mixture of EG 25% in the basal diet (as a feed) + AT 1% of the basal diet (as a feed additive), which achieved up to 40% CH_4_-reduction compared with their potential as individual supplementations: 11% for EG 25%, and 21.3% for AT 1%. Similarly, the combination EG 10% + AT 1% had a slight synergistic effect, leading to a 29.9% reduction in CH_4_ production.

The anaerobic fermentation of feed by rumen microorganisms results in the production of VFAs, which are the major energy sources for ruminant animals [55]. Any dietary intervention that can lead to the inhibition of methanogenesis at the expense of decreased VFA production is undesirable. The effect of AT supplementation on total VFA production varies in the literature, with some authors reporting a decrease [21], while others report no effect [20,56]. The main reason for this discrepancy is attributed to the dosages used and the bromoform concentration [47]. In the current study, AT supplementation at an effective dosage of 2.5% (0.112 mg bromoform/g DM feed incubated) with 80% CH_4_-reduction potential had no significant adverse effect on total VFA production, which might be related to the concentration of bromoform being lower than that reported in other studies where bromoform was 6.6 mg/g [20] and 7.8 mg/g [45]. Adding AT favored the fermentation pathway toward propionogenesis, which has been considered as a major hydrogen sink alternative to methanogenesis. This finding was also observed in other studies that evaluated the impact of AT on the fermentation profile [20,47,56]. The production of total VFA was not adversely affected by EG up to 25% inclusion. Although there was a CH4-reduction with EG inclusion, the concentration of propionate did not increase. This may indicate that the propionogenic pathway is not the reason for the inhibition of CH_4_. Therefore, decreasing the fermentation rate by inhibiting cellulolytic bacteria, resulting in less hydrogen and CO_2_ being available for methanogenesis, might be the explanation for the inhibition of CH_4_ in this study. This theory is further supported by the lower production CO_2_ with increasing EG levels. The IVDMD was not affected by the effective level of AT, as the fermentation rate was not inhibited. The increased IVDMD with EG inclusion might be due to the lower fiber content and higher content of digestible nutrients, as reported previously [17,57]. However, this is contradictory to the production of total VFA in the current study, which is the same as that observed in a previous in vitro trial conducted by Aemiro et al. [17]. Another possible explanation for the increased IVDMD could be the loss of some fine EG particles from the nylon bag during the incubation and washing procedures. Therefore, to confirm this theory, further experiments must be conducted with bags with smaller pore sizes to ensure that there is no loss of EG from the bag. It is noteworthy that digestibility-associated parameters for EG as a feed should be interpreted with caution; therefore, we only considered yield (mL/g) of CH_4_/DM in our comparisons. The higher concentration of NH_3_-N with EG 25% might be attributed to the high CP content of EG, which may have stimulated the activity of proteolytic rumen microbes. This nitrogen source can be utilized as a precursor for amino acids and for the synthesis of microbial proteins [58].

## 5. Conclusions

The current study demonstrated the high nutritive value of EG as an alternative high-quality feed to replace concentrate up to a 25% inclusion level in a ruminant diet without adverse effects on ruminal fermentation characteristics. This strategy was accompanied by a reduction in the CH_4_ yield of up to 11%. The AT harvested from Japan, when supplemented to a ruminant diet as a feed additive at 1 and 2.5% of the basal diet, led to a CH_4_-reduction of up to 21 and 80%, respectively. As a novel intervention, the combination of 25% EG as a feed and 1% AT as a feed additive had a synergistic effect, reducing CH_4_ production by up to 40%. This intervention has double-sided benefits, providing high-quality alternative feed and reducing CH_4_ with less AT. This study should be the first step toward further in vivo trials to ensure the efficacy of the mixture.

## Figures and Tables

**Table 1:** Chemical composition (% in dry matter) used for 24 h in vitro incubation.

%	Kleingrass Hay	Concentrate Mixture	*Euglena gracilis*	*Asparagopsis taxiformis*
Organic matter	89.19	93.73	95.56	41.74
Crude ash	10.81	6.27	4.44	58.26
Crude protein	14.58	18.83	26.10	13.28
Ether extract	3.68	4.32	17.65	2.31
Neutral detergent fiber	62.52	31.48	0.60	21.99
Acid detergent fiber	33.82	11.69	0.50	9.16
Acid detergent lignin	6.22	2.54	0.00	4.59
Non-fiber carbohydrates	8.41	39.1	51.21	4.16
Ingredients of the concentrate mixture (%)				
Corn		41.0		
Wheat		5.0		
Soybean meal		14.0		
Rapeseed meal		11.0		
Corn gluten		17.0		
Dried distiller’s grains with solubles		7.0		
Wheat bran		1.0		
Molasses		1.5		
Calcium carbonate		1.5		
Vitamin and mineral complex		1.0		

**Table 2:** Amino acids profile (%) of *Euglena gracilis*.

Amino Acid	% In Amino Acid Profile
Arginine	6.99
Lysine	7.28
Histidine	2.74
Phenylalanine	4.78
Tyrosine	4.20
Leucine	8.69
Isoleucine	4.20
Methionine	2.29
Valine	6.69
Alanine	7.44
Glycine	5.20
Proline	6.32
Glutamic acid	11.98
Serine	4.24
Threonine	4.99
Asparagine	8.48
Tryptophan	1.87
Cysteine	1.62

**Table 3:** Fatty acids profile (%) of *Euglena gracilis*.

Fatty Acid	% In Fatty Acid Profile
10:0	0.5
12:0	7.4
13:0	8.0
14:0	41.0
14:1	0.4
15:0	2.5
16:0	8.8
16:1	1.7
16:3	0.9
17:0	0.5
17:1	0.8
18:0	1.5
18:1	3.9
18:2n − 6	1.7
18:3n − 3	1.1
20:1	0.2
20:2n − 6	1.6
20:3n − 6	3.1
20:3n − 3	0.2
20:4n − 6	2.9
20:4n − 3	1.0
20:5n − 3	0.6
22:4n − 6	2.6
22:5n − 6	0.9
22:5n − 3	0.2
unknown	6.0

**Table 4:** Chemical composition (% in dry matter) of the experimental treatments with the combination of *Euglena* and *Asparagopsis*.

%	Control	*Euglena gracilis*		*Asparagopsis taxiformis*		*Euglena gracilis* (EG) + *Asparagopsis taxiformis* (AT)			
	0%	10%	25%	1%	2.5%	EG 10% + AT 1%	EG 10% + AT 2.5%	EG 25% + AT 1%	EG 25% + AT 2.5%
Dry matter	89.24	90.03	91.23	89.24	89.26	90.03	90.04	91.22	91.20
Organic matter	91.46	91.64	91.92	90.97	90.25	91.15	90.43	91.42	90.69
Crude ash	8.54	8.36	8.08	9.03	9.75	8.85	9.57	8.58	9.31
Crude protein	16.71	17.43	18.52	16.67	16.62	17.39	17.33	18.47	18.39
Ether extract	4.00	5.33	7.33	3.98	3.96	5.30	5.26	7.28	7.21
Neutral detergent fiber	47.00	43.91	39.28	46.75	46.39	43.69	43.38	39.11	38.86
Acid detergent fiber	22.76	21.64	19.96	22.62	22.42	21.51	21.33	19.85	19.69
Acid detergent lignin	4.38	4.13	3.75	4.38	4.39	4.13	4.14	3.75	3.77
Non-fiber carbohydrates	23.76	24.97	26.78	23.56	23.28	24.76	24.46	26.56	26.23

**Table 5:** Effect of *Euglena*, *Asparagopsis*, and their combination on gas production profile from 24 h in vitro incubation (n = 12).

	Treatments ^1^										Polynomial Contrast		Contrasts between Treatments		
Parameter	Control	AT 1%	AT 2.5%	EG 10%	EG 25%	EG 10% + AT 1%	EG 10% + AT 2.5%	EG 25% + AT 1%	EG 25% + AT 2.5%	SEM	AT	EG	AT × AT + EG	EG × AT + EG	AT 1% × AT 1% + EG (10% and 25%)
Total Gas/DM ^2^ (mL/g)	123.99 ^ab^	125.32 ^a^	117.88 ^abc^	118.91 ^abc^	109.24 ^cd^	114.36 ^bc^	109.21 ^cd^	102.60 ^de^	93.15 ^e^	1.46	Ns	L	<0.001	<0.001	<0.001
Total gas/DMD ^3^ (mL/g)	217.02 ^a^	208.32 ^ab^	201.82 ^bc^	187.44 ^cd^	157.41 ^e^	188.78 ^cd^	175.13 ^d^	148.20 ^ef^	139.57 ^f^	2.83	L	L	<0.001	0.003	<0.001
CH_4_ (%)	7.32 ^a^	5.69 ^b^	1.46 ^c^	7.29 ^a^	7.38 ^a^	5.55 ^b^	1.93 ^c^	5.27 ^b^	1.70 ^c^	0.24	L Q	Ns	0.89	<0.001	0.33
CO_2_ (%)	92.68 ^c^	94.32 ^b^	98.54 ^a^	92.71 ^c^	92.62 ^c^	94.45 ^b^	98.07 ^a^	94.73 ^b^	98.30 ^a^	0.24	L Q	Ns	0.89	<0.001	0.33
CH_4_/DM (mL/g)	9.11 ^a^	7.17 ^bc^	1.81 ^d^	8.71 ^a^	8.11 ^ab^	6.39 ^c^	2.20 ^d^	5.46 ^c^	1.66 ^d^	0.31	L	Ns	0.089	<0.001	0.01
CH_4_/DMD (mL/g)	15.92 ^a^	11.86 ^c^	2.97 ^e^	13.71 ^b^	11.71 ^bc^	10.51 ^c^	3.45 ^e^	7.88 ^d^	2.51 ^e^	0.49	L	L	0.004	<0.001	<0.001
CO_2_/DM (mL/g)	114.88 ^ab^	118.15 ^a^	116.07 ^ab^	110.21 ^abc^	101.13 ^cd^	107.97 ^b^	107.00 ^bc^	97.14 ^d^	91.49 ^d^	1.29	Ns	L	<0.001	0.02	<0.001
CO_2_/DMD (mL/g)	201.10 ^a^	196.46 ^a^	198.85 ^a^	173.73 ^b^	145.71 ^c^	178.26 ^b^	171.68 ^b^	140.32 ^cd^	137.06 ^d^	2.60	Ns	L	<0.001	0.22	<0.001

^1^ EG: *Euglena gracilis*; AT: *Asparagopsis taxiformis*. ^2^ DM: Dry matter. ^3^ DMD: Dry matter degraded. SEM: Standard error of the mean. ^a–f^ Values with different superscripts in the same row are significant different among different groups by Tukey test (*p* < 0.05). L: Linear (*p* < 0.05); Q: Quadratic (*p* < 0.05); Ns: Not significant (*p* > 0.05), by orthogonal polynomial contrasts.

**Table 6:** Effect of *Euglena*, *Asparagopsis*, and their combination on ruminal fermentation characteristics from 24 h in vitro incubation (n = 12).

	Treatments ^1^										Polynomial Contrast		Contrasts between Treatments		
Parameter	Control	AT 1%	AT 2.5%	EG 10%	EG 25%	EG 10% + AT 1%	EG 10% + AT 2.5%	EG 25% + AT 1%	EG 25% + AT 2.5%	SEM	AT	EG	AT × AT + EG	EG × AT + EG	AT 1% × AT 1% + EG (10% and 25%)
pH	6.58 ^ab^	6.56 ^bc^	6.53 ^c^	6.58 ^ab^	6.60 ^a^	6.58 ^ab^	6.56 ^bc^	6.58 ^ab^	6.57 ^ab^	0.01	Ns	Ns	0.20	0.35	0.57
IVDMD ^2^ (%)	57.05 ^de^	60.20 ^cde^	58.28 ^de^	63.54 ^bc^	70.27 ^a^	60.51 ^cde^	62.39 ^bcd^	69.23 ^a^	67.02 ^ab^	0.60	Ns	L	<0.001	0.03	0.001
Acetate (mmol/L)	257.13 ^a^	255.80 ^a^	236.84 ^b^	258.46 ^a^	255.00 ^a^	252.30 ^a^	237.40 ^b^	248.12 ^a^	228.37 ^b^	4.78	L	Ns	0.38	0.002	0.40
Propionate (mmol/L)	59.83 ^b^	65.85 ^ac^	68.75 ^a^	59.54 ^b^	55.92 ^d^	63.31 ^bc^	66.94 ^a^	60.19 ^b^	60.64 ^b^	0.80	L	L	0.002	<0.001	0.04
Butyrate (mmol/L)	23.59 ^bc^	24.73 ^ac^	25.70 ^a^	24.29 ^ab^	23.31 ^b^	24.52 ^ab^	25.46 ^a^	23.24 ^b^	23.63 ^bc^	0.29	Ns	Ns	0.18	0.55	0.38
Total VFA ^3^ (mmol/L)	340.56 ^ab^	346.38 ^a^	331.30 ^b^	342.29 ^ab^	334.23 ^ab^	340.13 ^ab^	329.79 ^b^	331.55 ^b^	312.64 ^c^	5.61	Ns	Ns	0.16	0.15	0.27
TVFA / DMD ^4^ (mol/g)	1.34 ^a^	1.29 ^a^	1.27 ^ab^	1.20 ^bc^	1.03 ^d^	1.24 ^b^	1.16 ^c^	1.05 ^d^	1.02 ^d^	0.02	Ns	L	<0.001	0.88	0.002
Acetate (mol/100 mol)	75.27 ^b^	73.57 ^d^	71.13 ^f^	75.23 ^bc^	76.03 ^a^	73.91 ^cd^	71.68 ^f^	74.57 ^c^	72.79 ^e^	0.26	L	L Q	<0.001	<0.001	0.01
Propionate (mol/100 mol)	17.71 ^e^	19.18 ^cd^	20.99 ^a^	17.59 ^e^	16.91 ^f^	18.78 ^d^	20.50 ^a^	18.33 ^d^	19.59 ^c^	0.18	L	L	<0.001	<0.001	0.001
Butyrate (mol/100 mol)	7.03 ^d^	7.25 ^bcd^	7.87 ^a^	7.17 ^bcd^	7.06 ^bcd^	7.32 ^bc^	7.82 ^a^	7.10 ^cd^	7.62 ^a^	0.09	L	Ns	0.36	<0.001	0.78
A/P ^5^ ratio	4.27 ^b^	3.86 ^d^	3.42 ^e^	4.31 ^b^	4.52 ^a^	3.96 ^d^	3.52 ^e^	4.10 ^c^	3.76 ^d^	0.05	L	L	<0.001	<0.001	0.001
NH_3_-N ^6^ (mg/dL)	8.73 ^c^	8.16 ^c^	8.54 ^c^	9.58 ^bc^	11.52 ^ab^	8.50 ^c^	8.48 ^c^	11.87 ^a^	10.83 ^ab^	0.50	Ns	Ns	0.18	0.59	0.22

^1^ EG: *Euglena gracilis*; AT: *Asparagopsis taxiformis*. ^2^ IVDMD: In vitro dry matter digestibility. ^3^ VFA: Volatile fatty acids. ^4^ DMD: Dry matter degraded. ^5^ A/P: Acetate/Propionate. ^6^ NH_3_-N: ammonia-nitrogen. SEM: Standard error of the mean. ^a–f^ Values with different superscripts in the same row are significantly different among different groups by Tukey test (*p* < 0.05). L: Linear (*p* < 0.05); Q: Quadratic (*p* < 0.05); Ns: Not significant (*p* > 0.05) by orthogonal polynomial contrasts.

## Data Availability

The datasets supporting reported results in this study are available on reasonable request from the corresponding author.
